# Pelvic Hydatid Cyst with Hydroureteronephrosis: A Rare Case Report

**DOI:** 10.1155/2021/2090849

**Published:** 2021-12-28

**Authors:** Amir Mohammad Salehi, Hossain Salehi, Ensiyeh Jenabi

**Affiliations:** ^1^School of Medicine, Hamadan University of Medical Sciences, Hamadan, Iran; ^2^Gastroenterology Ward, Baharlo Hospital, Tehran University of Medical Sciences, Tehran, Iran; ^3^Autism Spectrum Disorders Research Center, Hamadan University of Medical Sciences, Hamadan, Iran

## Abstract

Hydatid cyst is a parasitic disease caused by *Echinococcus granulosus* or *Echinococcus multilocularis*. Humans are accidentally infected with the parasite. The cyst is usually found in the liver and lungs and rarely occurs in other body parts. The present article describes a rare case of pelvic hydatid cyst in a young man who presented with nausea, vomiting, and right abdominal pain. Two large cystic masses were discovered during a CT scan in the patient's pelvic region, resulting in right urinary tract hydroureteronephrosis. Additionally, the antibody index was used to confirm the presence of a primary hydatid cyst.

## 1. Introduction

The hydatid cyst is a significant zoonotic disease caused by *Echinococcus granulosus* or *Echinococcus multilocularis* [1]. Humans are accidental hosts of this parasite, which they contract by eating the parasite's eggs. Infection occurs through direct contact with dog's stool [2]. The disease is prevalent worldwide, especially in the Mediterranean region, the Middle East, Australia, and New Zealand. Hydatid cysts are most frequently found in the liver (50–70%) and lungs (20–30%), but they can rarely occur in any organ, including the brain, heart, bone, abdomen, and pelvis [3, 4].

Peritoneal hydatidosis can be primary or secondary to hydatid cysts in the liver or, less frequently, the spleen. Primary peritoneal hydatidosis is uncommon, occurring in only 2% of all cases of abdominal hydatid disease [1]. Unusual cyst locations complicate and delay diagnosis, resulting in severe complications for the patient [5]. The present article reports a rare case of a primary hydatid cyst in the intraperitoneal pelvic space that caused hydroureteronephrosis due to its compressive effect on the ureter.

## 2. Case Description

In August 2021, a 33-year-old single man living in Hamadan complained of nausea, vomiting, and weakness over the previous three days, a lack of response to antinausea medication, severe right abdominal pain, and decreased urine volume. The patient was an insurance company employee who mentioned visiting villages near Hamedan approximately a month prior (July 2021), staying for ten days, and consuming the local cuisine. The patient was conscious during the physical examination. The physical examination revealed a body temperature of 37°C, a heart rate (HR) of 90 beats per minute, a respiratory rate (RR) of 15 beats per minute, and a blood pressure (BP) of 105/75 mmHg. The patient exhibited slight paleness, had no smoking history, and the heart sounds and lungs auscultation was unremarkable.

**Figure 1:**
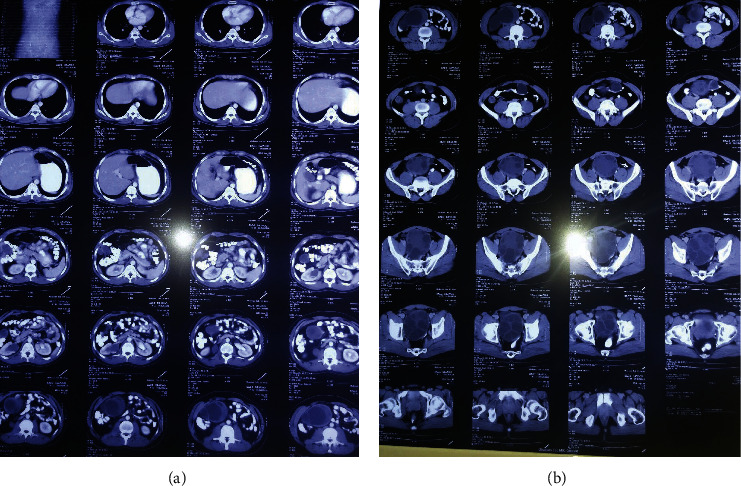
CT showing no involvement of the liver (a). CT showing a pelvic hydatid cyst with hydroureteronephrosis (b).

The patient demonstrated a right tenderness to the percussion of CV angle. Due to abdominal pain, the patient was admitted to the internal ward. Except for the leukocyte count (12000 with pmn 88%), all tests were normal, including the differential count, renal function tests, liver function tests, random blood sugar, and urinalysis. The patient requested abdominal and pelvic CT scans with IV and oral contrast. On CT, the patient had only two large cystic masses in the pelvic region, one of which was compressed against the right ureter, resulting in right urinary tract hydroureteronephrosis (Figures 1(a) and 1(b)).

**Figure 2:**
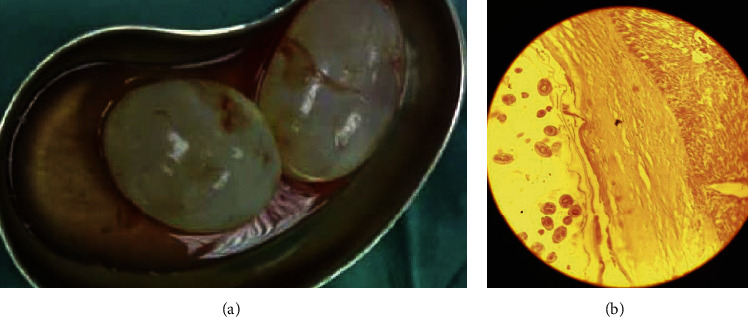
Cysts removed after surgery (a). The pathological specimen of the removed cysts, which included the chitinous layer and the germinal layer region in the incision (b).

A double J sonde was temporarily inserted on the right side of the urinary tract after consulting with the hospital urologist. The ELISA test was requested because there was a possibility of a hydatid cyst. The hydatid antibody index was 29 (normal index range below 11). The patient was given 400 mg of albendazole twice a day and referred to a surgeon. After two days of drug administration, the patient underwent exploratory laparotomy, which resulted in removing two cysts. The pathologist confirmed the hydatid cyst (Figures 2(a) and 2(b)). The patient was discharged from the hospital two days later and was prescribed albendazole for up to one month after surgery. After being discharged, he was referred for a six-month follow-up.

## 3. Discussion

Hydatid cystisa zoonotic disease was caused by *Echinococcus granulosus* or *Echinococcus multilocularis*. Humans become infected with this parasite accidentally. Infected dogs' feces carry the eggs, which infect and spread, and the eggs are resistant to drying and can survive for weeks in their living environment. As a result, they can infect a person without direct contact with the carrier animal. The parasite enters the human body through contaminated food or water that contains parasite eggs. Once the parasite's eggs are consumed, they enter the small intestine and travel through it to the veins and lymphatic vessels, where they spread to various organs of the body, including the liver and lungs, where they eventually develop into larval cysts [2, 4].

Hydatid cysts affect the liver and lungs in 59–75% and 27% of cases, respectively [6]. Other organs (the abdomen, pelvis, and nervous system) account for only 10% of cases [3]. Hydatid cyst disease is widespread worldwide, particularly in livestock-producing countries [3]. According to epidemiological studies, the prevalence of hydatid cysts in Iran is higher in women than in men. Furthermore, hydatid cysts are more common in rural communities in Iran. The majority of cases involved individuals between the ages of 30 and 49. Moreover, there has been an increase in cases with liver involvement [7, 8].

Cysts grow to a diameter of 5 to 10 cm in a year and may persist for years, if not decades. Symptoms are typically absent, and the infection is frequently detected accidentally during imaging for other purposes. Symptoms, if they occur, will be caused by the cyst's compressive effect in a confined space. Cysts in the abdomen and pelvis can also result in swelling and pain in the abdomen and pelvis [9]. The patient, in this case, presented with nausea, vomiting, and right side pain, as well as hypotension.

A hydatid cyst contains an inner germinal layer that asexually germinates daughter cysts within the original cyst. Cyst rupture has been linked to life-threatening allergic reactions to parasite antigens. The patient develops an anaphylactic reaction, including hypotension, syncope, and fever, following a ruptured cyst in the most severe case. A potentially dangerous complication of cyst rupture is the implantation of daughter cysts in other parts of the body, which results in organ failure and a significant increase in disability and mortality in patients [9].

Hydatid disease should be diagnosed using a combination of history, physical examination, laboratory tests, and imaging. CT and MRI are the most effective imaging techniques for cystic echinococcosis diagnosis. Serological tests and antigens specific for cystic echinococcosis are used in laboratory testing. Serological tests have a sensitivity of 80%–100% and a specificity of 88–96% in the case of liver cysts, but only 50%–60% in the case of lungs and 25%–56% in the case of other organs [10]. Imaging techniques remain more sensitive than serological tests, and a typical scan in the presence of negative serology still suggests a diagnosis of cystic echinococcosis. Complete surgical removal is the most appropriate treatment option for symptomatic cysts [9]. Due to hydroureteronephrosis, our patient's right kidney was surgically removed, which is the best treatment option for patients with hydatid cysts.

Albendazole and mebendazole are beneficial in cases where the patient is unable to undergo surgery, in cases of liver and lung cysts and in cases where more than two organs are involved [11]. Preoperative treatment with antiechinococcal agents such as albendazole and mebendazole to prevent the spread of daughter cysts during surgery appears to be a more cautious approach. Administering these drugs is proven to be more effective in young people than in the elderly [12]. Additionally, drug therapy alone has improved the condition of many patients (55–79%) and resulted in complete recovery in others (29%). Albendazole is administered in three or more doses and up to six doses at two-week intervals. The drug is given at 10–15 mg per kg of body weight per day for four weeks during each period. Each treatment period is followed by a week of rest without medication [12, 13]. Imaging techniques are the most effective way to monitor treatment response. A positive response to treatment is defined as the disappearance or shrinkage of the cyst combined with an increase in density [14].

## 4. Conclusion

This report presents a rare case of intraperitoneal hydatid cyst in which the patient complained of nausea, vomiting, and lethargy over the previous three days and an inability to respond to antinausea treatment severe rightside pain and decreased urine volume. Only 2% of intra-abdominal hydatid cysts are intra-abdominal. Thus, if a patient lives in rural areas and has a history of contact with dogs or sheep, a hydatid cyst should be considered as one of the differential diagnoses.

## Data Availability

The data used to support the findings of this study are available from the corresponding author on up request.

## References

[1] Parray F. Q., Wani S. N., Bazaz S., Khan S.-R., Malik N. S. (2011). Primary pelvic hydatid cyst: a case report. *Case reports in surgery*.

[2] Menezes da Silva A. (2003). Hydatid cyst of the liver-criteria for the selection of appropriate treatment. *Acta Tropica*.

[3] Ciurea A. V., Fountas K. N., Coman T. C. (2006). Long-term surgical outcome in patients with intracranial hydatid cyst. *Acta Neurochirurgica*.

[4] Ghafouri M., SeyedSharifi S. (2016). Pelvic hydatid cyst in a young woman; A rare case report. *Journal of Rafsanjan University of Medical Sciences*.

[5] Ben Rejeb C., Dhifallah S., Bibi M. (2001). Bilateral hydatid cyst of the fallopian tubes: a case report. *Journal de Gynecologie, Obstetrique et Biologie de La Reproduction*.

[6] Yuksel M., Demirpolat G., Sever A., Bakaris S., Bulbuloglu E., Elmas N. (2007). Hydatid disease involving some rare locations in the body: a pictorial essay. *Korean Journal of Radiology*.

[7] Moosazadeh M., Abedi G., Mahdavi S. A., Shojaee J., Charkame A., Afshari M. (2017). Epidemiological and clinical aspects of patients with hydatid cyst in Iran. *Journal of Parasitic Diseases*.

[8] Mahmoudi S., Mamishi S., Banar M., Pourakbari B., Keshavarz H. (2019). Epidemiology of echinococcosis in Iran: a systematic review and meta-analysis. *BMC Infectious Diseases*.

[9] Nazari Z., Torabizadeh J. (2014). Primary hydatid cyst of the fallopian tube: a case report. *Caspian journal of internal medicine*.

[10] Macpherson C. N. L., Milner R. (2003). Performance characteristics and quality control of community based ultrasound surveys for cystic and alveolar echinococcosis. *Acta Tropica*.

[11] Zhang W., Li J., McManus D. P. (2003). Concepts in immunology and diagnosis of hydatid disease. *Clinical Microbiology Reviews*.

[12] Junghanss T., Brunetti E., Chiodini P. L., Horton J., Da Silva A. M. (2008). Clinical management of cystic echinococcosis: state of the art, problems, and perspectives. *The American Journal of Tropical Medicine and Hygiene*.

[13] Kapan S., Turhan A. N., Kalayci M. U., Alis H., Aygun E. (2008). Albendazole is not effective for primary treatment of hepatic hydatid cysts. *Journal of Gastrointestinal Surgery*.

[14] Dziri C., Haouet K., Fingerhut A. (2004). Treatment of hydatid cyst of the liver: where is the evidence?. *World Journal of Surgery*.

